# Novel composite Zr/PBI-O-PhT membranes for HT-PEFC applications

**DOI:** 10.3762/bjnano.4.57

**Published:** 2013-08-21

**Authors:** Mikhail S Kondratenko, Igor I Ponomarev, Marat O Gallyamov, Dmitry Yu Razorenov, Yulia A Volkova, Elena P Kharitonova, Alexei R Khokhlov

**Affiliations:** 1 Faculty of Physics, Lomonosov Moscow State University, Leninskiye Gory 1-2, GSP-1, Moscow, 119991, Russia; 2 Nesmeyanov Institute of Organoelement Compounds, Russian Academy of Sciences, Vavilova St. 28 , GSP-1, Moscow, 119991, Russia

**Keywords:** composite, high temperature polymer-electrolyte fuel cells (HT-PEFC), impedance spectroscopy, polybenzimidazole (PBI), zirconium

## Abstract

Novel composite membranes for high temperature polymer-electrolyte fuel cells (HT-PEFC) based on a poly[oxy-3,3-bis(4′-benzimidazol-2″-ylphenyl)phtalide-5″(6″)-diyl] (PBI-O-PhT) polymer with small amounts of added Zr were prepared. It was shown in a model reaction between zirconium acetylacetonate (Zr(acac)_4_) and benzimidazole (BI) that Zr-atoms are capable to form chemical bonds with BI. Thus, Zr may be used as a crosslinking agent for PBI membranes. The obtained Zr/PBI-O-PhT composite membranes were examined by means of SAXS, thermomechanical analysis (TMA), and were tested in operating fuel cells by means of stationary voltammetry and impedance spectroscopy. The new membranes showed excellent stability in a 2000-hour fuel cell (FC) durability test. The modification of the PBI-O-PhT films with Zr facilitated an increase of the phosphoric acid (PA) uptake by the membranes, which resulted in an up to 2.5 times increased proton conductivity. The existence of an optimal amount of Zr content in the modified PBI-O-PhT film was shown. Larger amounts of Zr lead to a lower PA doping level and a reduced conductivity due to an excessively high degree of crosslinking.

## Introduction

Polymer-electrolyte fuel cells (PEFC) based on polybenzimidazole (PBI) membranes doped with phosphoric acid (PA) as an electrolyte can be operated without any humidification of reactant gases at an elevated temperature range, in which the CO tolerance of the Pt catalyst becomes higher. This allows the use of cheap hydrogen fuel, which was not thoroughly purified, such as hydrogen produced by natural gas reforming, at the place of consumption. On the way to commercialization, however, this promising type of PEFC still faces several problems concerning its long term stability and the overall efficiency of electrode and membrane materials. These properties should be further improved in order to make HT-PEFC economically reasonable.

The proton conductivity of PBI membranes generally increases with the PA doping level [1]. At the same time, higher PA doping levels usually result in decreased mechanical strength, which may lead to an increased crossover of reactant gases. The design of an advanced membrane for HT-PEFC applications necessitates the finding of an appropriate compromise between proton conductivity on the one hand and good mechanical properties as well as low gas permeability on the other hand.

Various PBI-based composites have been proposed in order to achieve an optimal balance of these properties. In order to enhance the mechanical strength, various polymers, such as PTFE [2–3] or polymer sulfonic acids, which can form ionic bonds with basic PBI (Nafion [4], SPEEK [5–6]), were proposed as functional fillers for PBI membranes. Even carbon nanotubes were impregnated into PBI matrices for a higher durability [7–8]. In order to improve the conductivity, proton conductors such as heteropolyacids (H_3_SiW_12_O_40_ (SiWA) [9–12], H_3_PW_12_O_40_ (PWA) [12], Cs_2.5_H_0.5_PMo_12_O_40_ (CsPMoA) [13]), lithium hydraziniumsulfate, LiN_2_H_5_SO_4_, (LiHzS) [14] and Zr-containing compounds (zirconium pyrophosphate [15], zirconium tricarboxybutylphosphonate [16–17]) were introduced into PBI membranes. According to the literature, researchers generally use quite high amounts (10–50%) of modifying agents when producing composite membranes. Such high amounts are required to achieve an optimal structure of the functional filler inside the PBI matrix, i.e., a well-developed proton-conducting channel system if solid proton conductors are added, or a durable polymer frame, e.g., for ionic crosslinking by polymer sulfonic acids.

**Figure 1:**
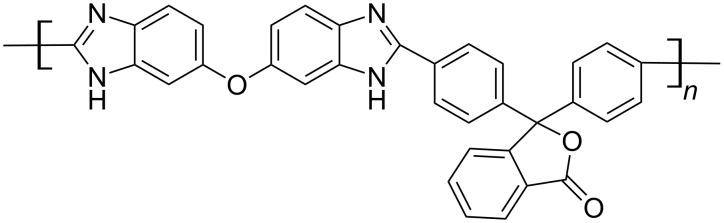
Chemical structure of poly[oxy-3,3-bis(4′-benzimidazol-2″-ylphenyl)phtalide-5″(6″)-diyl] (PBI-O-PhT).

In the present work we propose an alternative approach to the design of composite membranes based on PBI. We propose adding a rather small number of zirconium atoms into PBI matrices by means of Zr precursors (zirconium tetraacetate (Zr(OAc)_4_) or zirconium acetylacetonate (Zr(acac)_4_)). Zirconium atoms, which have a valence of four, exhibit coordination numbers of up to nine and may form crosslinks between PBI chains. In this way, they can improve the chemical and thermal stability as well as the mechanical strength of a membrane. At the same time, zirconium is able to form acidic phosphates with intrinsic proton conductivity, and acts as a coordination centre for PA, which improves the electrolyte binding in the matrix. This should result in increased proton conductivity, and a more effective transport and better acid retention in a membrane during long-time operation. For the present paper we examined the properties of composite membranes based on poly[oxy-3,3-bis(4′-benzimidazol-2″-ylphenyl)phtalide-5″(6″)-diyl] (PBI-O-PhT, chemical structure is shown in Figure 1 ) [18–19] with the addition of small amounts of zirconium. Additionally, the performance of HT-PEFC based on these composite membranes is studied.

## Experimental

### Membrane preparation

#### PBI-O-PhT synthesis

Initially, 3,3',4,4'-tetraaminodiphenyl ether (0.461 g, 2 mmol) and 4,4′-diphenylphtalidedicarboxylic acid (0.749 g, 2 mmol) were mixed under dry argon flow with 3.8 mL of Eaton’s reagent (P_2_O_5_:MeSO_3_H 9:1 wt.) in a three-neck flask equipped with a mechanical stirrer and a heater with temperature control. The mixture was stirred for 2 h at 80 °C, then for 1 h at 100 °C, and finally 1 h at 120 °C. Then 0.57 g (4 mmol) of P_2_O_5_ was added, and the reaction continued for a further 2 h at 120 °C. Then the temperature was raised to 145–150 °C, and the reaction continued for 2–5 h, until a dramatic increase in the viscosity of the mixture was observed. After that, the mixture was diluted with an equal volume of 85% H_3_PO_4_ and stirred to obtain a homogenous solution. The latter was slowly poured into water and dispersed, then filtered, washed with water until pH 7, treated with methanol for extraction of residuals in a Soxhlet extractor, and dried in vacuum for 5 h at 100 °C. The chemical structure of the product was confirmed by ^1^H NMR and IR spectra, and elemental analysis. The intrinsic viscosity, [η], measured in *N*-methylpyrrolidone (NMP) at 25 °C was 2.02 dL/g.

#### Film casting and crosslinking

**Standard procedure:** Polymer films were cast from a 10% polymer solution in NMP on glass plates heated at 60–80 °C. After solvent evaporation (8–12 h), the films were heated in vacuum at 160 °C for 2 h for additional drying, put in hot water to extract any residuals, then placed in 2% H_2_SO_4_ for 24 h at room temperature, and then heated in an oven with air circulation for 1 h at 350 °C for the three-dimensional crosslinking of polymer chains.

**Zr-procedure:** Zr(IV) acetylacetonate (Zr(acac)_4_) or Zr(IV) tetraacetate (Zr(OAc)_4_) were dissolved in NMP and added to the polymer solution in corresponding quantities before casting. The subsequent procedure was the same as the standard one except for the step of immersing the films in 2% H_2_SO_4_.

**Doping with PA:** In order to obtain the membrane material, the cross-linked films were doped with 77% PA at 60 °C for three days. The resulting membrane thickness was about 50 μm. Before assembling the fuel cells, the membranes were stored in 85% PA at room temperature. In total, four series of membranes were prepared and tested: (1) Non modified PBI-O-PhT standard (reference membrane), (2) PBI-O-PhT modified by adding 0.75 wt % Zr(acac)_4_, (3) PBI-O-PhT modified by adding 2 wt % Zr(acac)_4_, and (4) PBI-O-PhT modified by adding 0.75 wt % Zr(OAc)_4_.

### SAXS

High resolution small-angle diffraction patterns of PBI-O-PhT and Zr/PBI-O-PhT composite membranes doped with PA were recorded with a SAXS- and WAXS camera S3-Micropix, manufactured by Hecus (Cu Kα, λ = 1.542 Å). Two detectors were used: a two-dimensional Pilatus 100K and a linear position-sensitive-detector PSD 50M operating at a pressure of 8 bar Ar/Me. A Xenocs Genix generator supplied the high-voltage (50 kV) and the current (1mA) for the detectors. Fox 3D vacuum optics were used to shape the X-ray beam, and the slits in the Kratky collimator were set to 0.1 and 0.2 mm, respectively. The angular scale was between 0.003 Å^−1^ and 1.9 Å^−1^. In order to eliminate the influence of air, the X-ray optics system and the camera were evacuated to a pressure of (2–3) × 10^−2^ mmHg. The exposition time was varied from 600 to 5000 s.

### FT-IR

Because the loading of Zr precursors in the composite membranes is very low, no noticeable changes in the FT-IR spectra of composite and reference samples could be observed. Hence, in order to confirm the active role of zirconium in the PBI-O-PhT crosslinking process we studied a model reaction of benzimidazole (BI) with Zr(acac)_4_ in a melt in the temperature range of 320–350 °C, which was also applied for heating the films. As a result of this interaction, a dark brown non-melting insoluble product of polymeric nature is formed. A mixture of BI with Zr(acac)_4_ (4:1 molar ratio) and the reaction product, which occurred after heating this mixture up to 320–350 °C, were pressed into KBr pellets, and FT-IR spectra were taken by using a Nicolet Magna-IR-750 spectrometer.

### TMA

Thermomechanical analysis of the composite membranes doped with PA was conducted by using a Netzsch TMA 202 instrument. Five heating/cooling cycles from 40 to 190 °C in air and under a constant load of 0.05 N were performed for each membrane sample.

### Fuel cell testing

#### Fuel cell assembly

Cells with an active area of 5 cm^2^ were assembled. Gas diffusion electrodes containing 1 mg·cm^−2^ Pt (Pt/C ratio = 40%) were taken from a commercial membrane–electrode assembly (MEA) Celtec P1000 (BASF). In our experience, they have a rather reproducible performance. Therefore, we used them in order to ensure the most reliable comparison of different membranes in operating fuel cells. Membranes were prepared according to the procedure described above. The membrane–electrode assemblies (MEAs) were assembled with fuel cell hardware units (Arbin Instruments) including bipolar graphite plates with a reagent supply system and current collectors.

#### Break-in

After assembly the fuel cells were heated up to 160 °C and operated at a constant current density of 0.4 A·cm^−2^ for a 50 hour break-in. Pure hydrogen and air were supplied separately to the anode and cathode electrodes, respectively, without any humidification or excessive pressure. The gas flows were controlled by Bronkhorst El-Flow mass-flow controllers, which used an RS-232 interface. During the electrochemical measurements the air flow on the cathode was kept at a rather high value of 200 mL·min^−1^ (corresponding to a stoichiometry of about 6 for a current density *j* = 0.4 A·cm^−2^) to minimize the oxygen-transport limitations [19].

#### Steady state polarization curves

All electrochemical measurements were performed by the use of an Autolab PGSTAT 302 (Eco Chemie) potentiostat/galvanostat with a built-in frequency response analyser module FRA 2. Steady state galvanostatic polarization curves were measured in a current density range from 0 to 0.4 A·cm^−2^ at 160 °C. The current step was 2 mA·cm^−2^. After setting each current value the system was allowed to reach a steady state for about 10 s before measuring the voltage.

#### Impedance measurements

**Figure 2:**
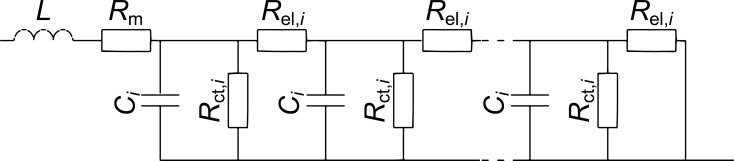
Equivalent circuit with a transmission line for modelling the impedance response of the active layers.

The impedance of the fuel cells with membranes of different types was measured in a galvanostatic mode at frequencies from 100 kHz to 0.1 Hz for ten different current density values (from 0.04 to 0.40 A·cm^−2^). The magnitude of the current perturbation was 2 mA·cm^−2^. After setting each direct-current density value, the system was allowed to reach a steady state for 10 min before taking the frequency scan. An equivalent circuit with a transmission line of *n* repeating units (Figure 2) was used for fitting the impedance data. Such an approach allows accounting for a distributed structure of active layers (AL) and is described in detail in our previous paper [19]. Each repeating unit with index *i* stands for a thin sublayer of the AL and models the following processes: charge-transfer during the oxygen reduction reaction, double layer charging and ohmic losses due to finite proton conductivity of the AL. The following parameters were obtained as a result of the impedance spectra approximation: the undistributed ohmic resistance of a cell, *R*_m_, (mainly membrane resistance); the distributed resistance of proton transport in the cathode AL, R_el_; the charge transfer resistance, R_ct_; and the double layer capacitance, *C* (Figure 2). Fitting of the impedance spectra was performed by means of the Zview modelling software using the DX-6 distributed element for the transmission line with *n* repeating units. In order to define the membrane resistances more accurately, the resistance of the test cell itself (including the resistance of current collecting plates, contacts and wires) was subtracted from the undistributed ohmic resistance, *R*_m_, obtained from the impedance data. In order to determine the resistance of the test cell we measured the resistance of the cell assembled without a membrane and with direct contact between the electrodes. The value of this resistance was 4.7 mΩ.

#### Hydrogen crossover-current measurements

For hydrogen crossover-current measurements, the cathode was fed with pure nitrogen, and pure hydrogen was supplied to the anode. The dry gases were supplied at an ambient pressure at flow rates of 50 mL·min^−1^. After several minutes the open-circuit voltage reached its steady-state value of about 120 mV. Then the voltage was swept slowly (1 mV·s^−1^) to 500 mV and the resulting current of hydrogen oxidation was recorded. A similar technique of hydrogen crossover-current measurements is described in [20–21].

#### 2000 hour durability test

PBI-O-PhT modified by adding 0.75 wt % Zr(acac)_4_ and a non-modified PBI-O-PhT reference membrane were tested in fuel cells operating at 160 °C at a constant current density of 0.4 A·cm^−2^ for 2000 h. Hydrogen and air flows were the same as described above.

## Results and Discussion

**Figure 3:**
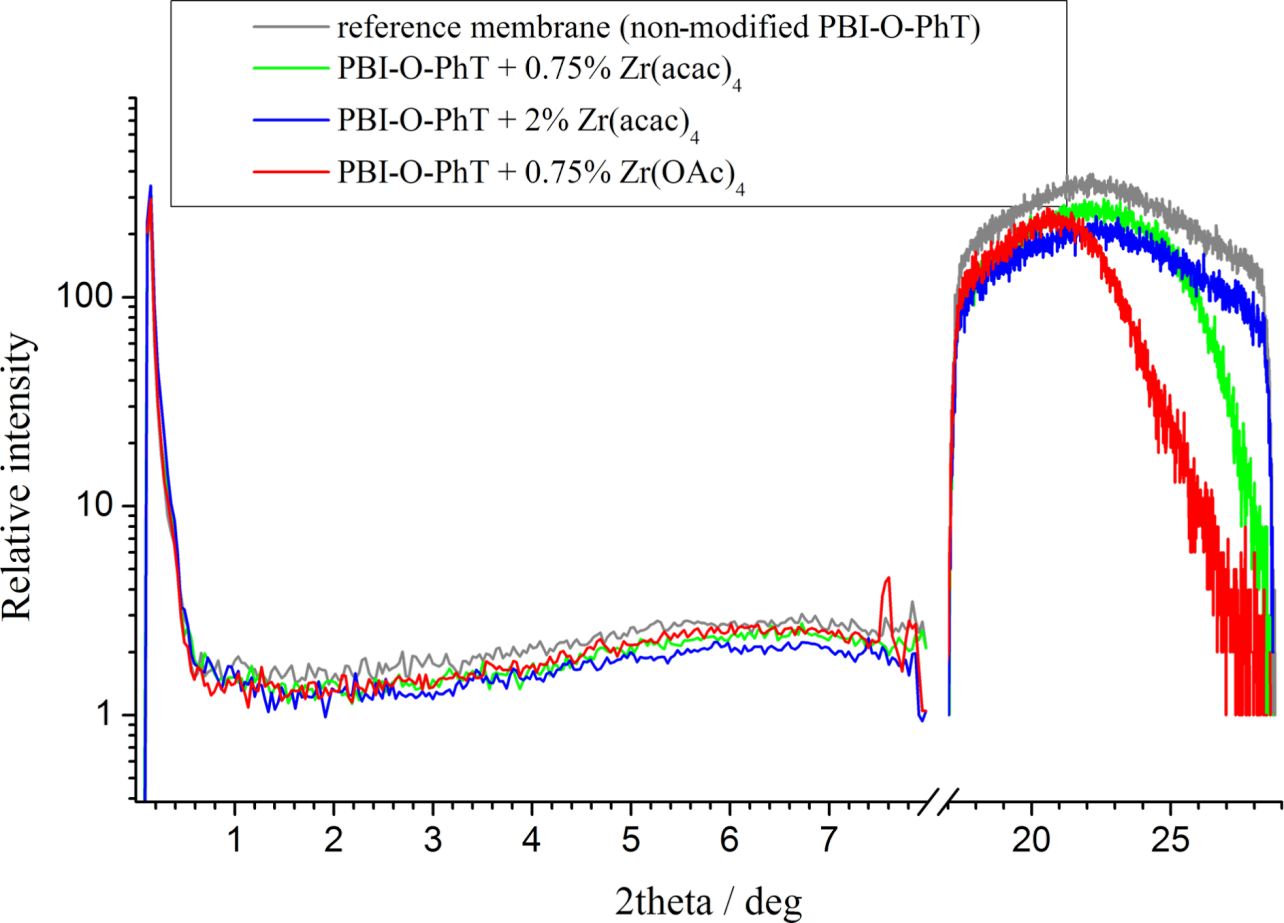
Small angle X-ray scattering results for the different types of composites and the reference membrane.

X-ray scattering data presented in Figure 3 indicate that the composite membranes have a uniform amorphous structure. Compared to the non-composite reference sample there are no noticeable differences. Similar SAXS patterns for PBI films have also been reported by Kannan et al. [8]. Only an amorphous halo without any scattering peaks is observed in the region between 20 and 30° (2θ) for all samples of the series. The absence of any noticeable structural changes in the composite membranes is quite expectable since we added only very small amounts of Zr compounds. According to the obtained data, Zr-based crystallites are not formed (the samples are amorphous), so that Zr atoms should be uniformly distributed inside the PBI-O-PhT films. Slight differences of the intensity of the scattered radiation above 25° are observed for membranes with different amounts of the Zr precursor. Membranes with higher Zr amounts (2 wt % Zr(acac)_4_) and the reference membrane have stronger structure correlations with a characteristic length of approximately 7 Å in comparison to membranes with lower Zr amounts (0.75 wt % Zr(acac)_4_ or Zr(OAc)_4_). This is probably explained by the different number of crosslinks, which is higher between polymer chains of the reference membrane and the membranes with 2 % Zr(acac)4 loadings than between polymer chains of the membranes with 0.75 % Zr precursors loadings.

**Figure 4:**
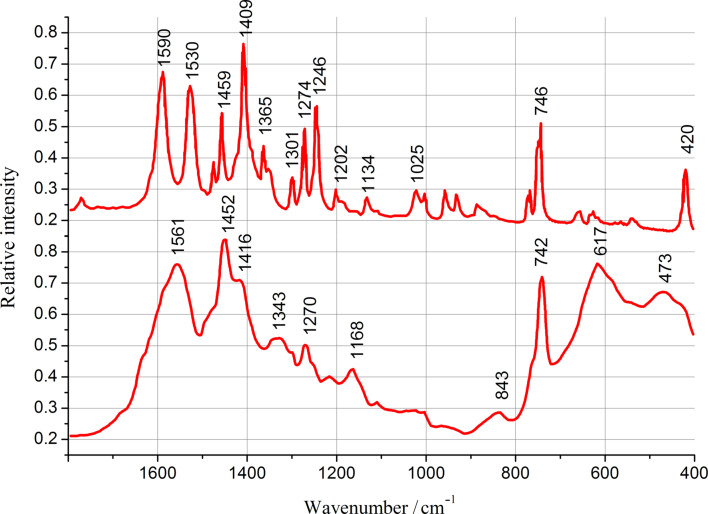
FT-IR spectra of a mixture of BI with Zr(acac)_4_ (4:1 molar ratio, upper spectrum) and of the product of the reaction occurring after heating of this mixture to 320–350 °C (lower spectrum).

In order to prove the active role of zirconium compounds in the PBI-O-PhT crosslinking process we studied a model reaction of benzimidazole (BI) with Zr(acac)_4_. FT-IR spectra of a mixture of BI with Zr(acac)_4_, and of the product of the reaction occurring after heating this mixture to 320–350 °C, are presented in Figure 4. The spectrum of the mixture after heating dramatically differs from the initial one. One can observe a noticeable shift and widening of the absorption peaks of the BI aromatic system at 1590, 1530, 1409, 1246 cm^−1^, and an appearance of several new strong broad peaks at 1561, 1452, 617, 473 cm^−1^, which can be attributed to the formation of both chemical and coordination bonds of zirconium with BI. According to this data one can conclude that the PBI-O-PhT macromolecules inside the membranes, which have been modified by adding Zr(acac)_4_ or Zr(OAc)_4_ and subsequent heating, are crosslinked by zirconium. It is noteworthy, that pristine non-crosslinked PBI-O-PhT films dissolve in PA. Adding small amounts of Zr makes the films stable and insoluble in PA even at 180 °C.

**Figure 5:**
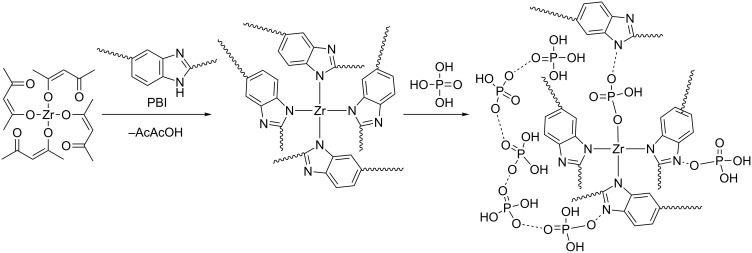
Possible mechanism of the crosslinking process of PBI by Zr(acac)_4_ and further doping with PA.

A possible mechanism of the crosslinking process of PBI by Zr(acac)_4_ and further doping with PA is shown in Figure 5. Due to lability of the N–Zr bonds in the excess of PA, one can expect a dynamical behaviour of the Zr-crosslinks. We suggest that zirconium is forming chemical bonds not only with PBI-O-PhT macromolecules but also with PA molecules after the doping procedure, as shown in the right part of Figure 5. Since the coordination number of zirconium ranges from six to nine, one can also expect coordination bonds of Zr atoms with several PA molecules (not shown in Figure 5). Due to the dynamic nature of the Zr-crosslinks and the coordination bonds of Zr with PA, the composite Zr/PBI-O-PhT membranes should show higher PA doping levels in comparison to the reference PBI-O-PhT membrane with stiff sulfuric crosslinks [18–19]. This increased acid uptake of the composite membranes is observed experimentally, and the results are presented in Table 1.

**Table 1:** Equilibrium phosphoric acid (PA) doping levels for composite PBI-O-PhT membranes.

membrane type	equilibrium PA doping level

reference membrane (pristine sulfuric-crosslinked PBI-O-PhT)	380%
PBI-O-PhT + 0.75 wt % Zr(acac)_4_	430%
PBI-O-PhT + 0.75 wt % Zr(OAc)_4_	430%
PBI-O-PhT + 2 wt % Zr(acac)_4_	400%

From Table 1 one can see that the transition from stiff sulfuric crosslinks in the reference PBI-O-PhT membrane (for comparison here we used pristine PBI-O-PhT membranes, thermally crosslinked in the presence of sulfuric acid as described in [18]) to dynamic crosslinks of PBI-O-PhT chains by zirconium resulted in a raise of acid uptake from 380 to 430% for samples with 0.75 wt % Zr precursor loading. An increase of this loading to 2 wt % leads to a lower acid uptake of 400% which can be explained by an excessive degree of crosslinking.

The strong tendency of Zr-atoms to coordinate atoms of both PBI chains and PA is rather fortunate not only from the viewpoint of enhanced mechanical properties of the composite, but also due to the expected improved ability to retain the liquid electrolyte. Indeed, in a typical PBI material suitable proton conductivity is achieved only at a doping level of several phosphoric acid molecules per PBI monomer unit. Only one PA molecule is really bound to the protonated N-atom, the other molecules are retained by hydrogen bonds. This acid–base bonding requires an immobilized proton to be excluded from the proton transport. In contrast, the direct coordination bonding between Zr and the O-atom of a PA molecule spares the corresponding proton for proton transport, but the additional contribution to hydrogen bonding for electrolyte molecules in the matrix is still achieved.

**Figure 6:**
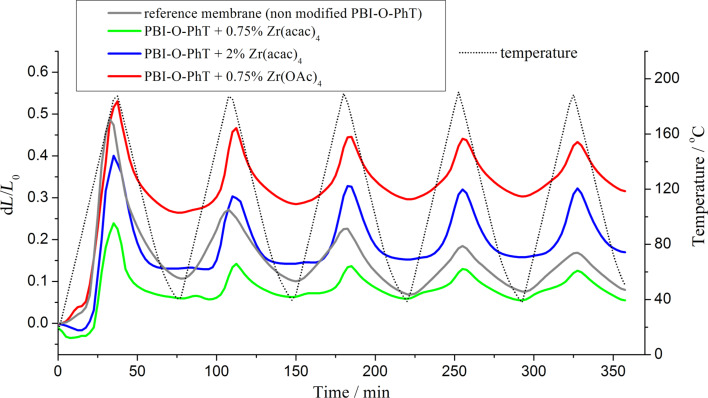
Change of the relative membrane thickness in a series of five consecutive heating/cooling cycles. The temperature change is given by the dotted line.

**Figure 7:**
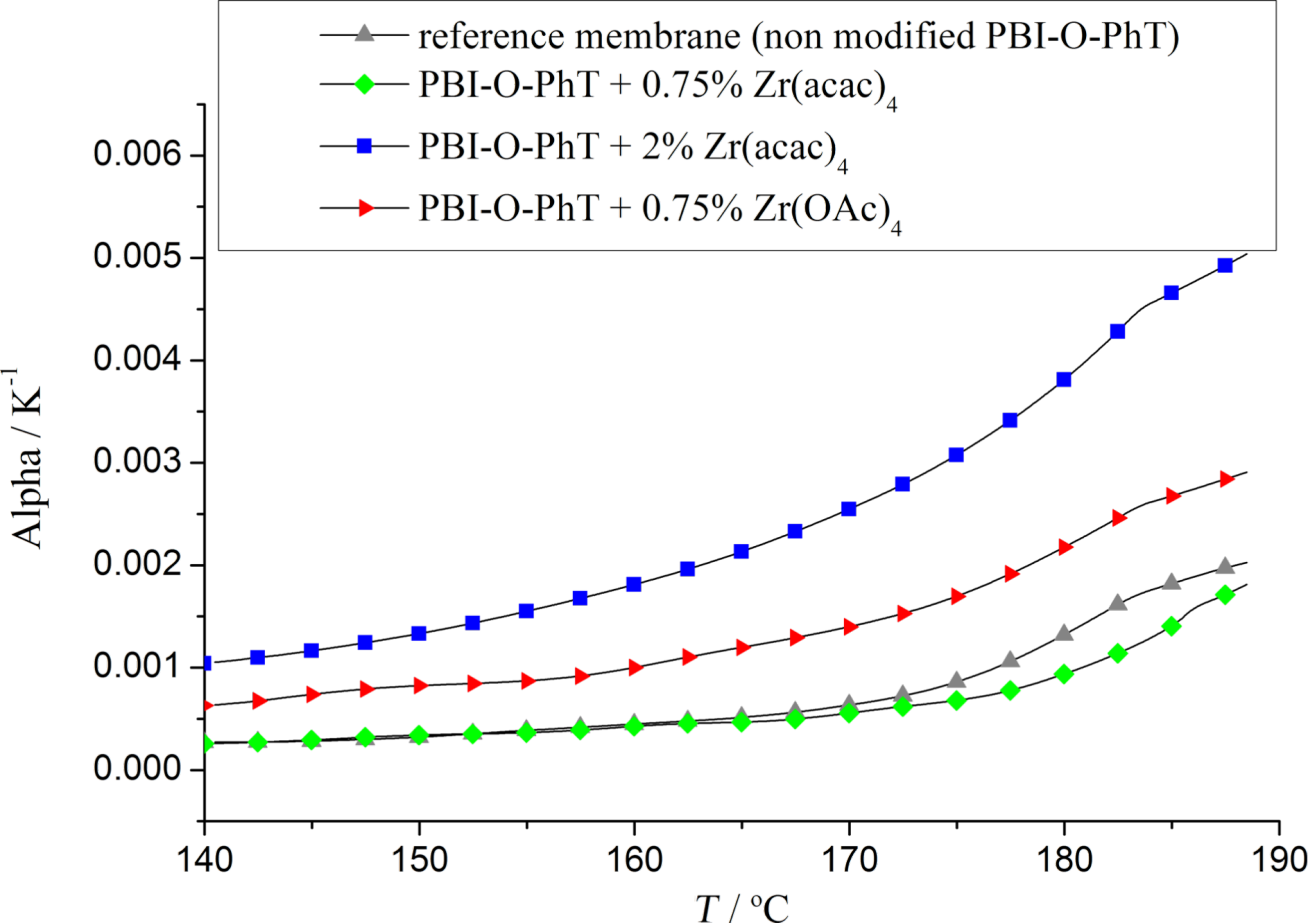
Thermal expansion coefficients of the composite membranes.

Composite membranes with Zr-crosslinks show a high acid uptake and, at the same time, demonstrate excellent mechanical stability in a temperature range of 20–190 °C, which is confirmed by TMA results presented in Figure 6 and Figure 7. The thermal expansion under constant load is smooth and can be reproduced well for several repeated heating/cooling cycles for all samples (Figure 6). The thermal expansion coefficient calculated from TMA data as presented in Figure 7 is positive and has similar values for the reference samples and the composites with 0.75 wt % loading of Zr precursors. Composite membranes with 2 wt % Zr(acac)_4_ have a higher crosslinking degree resulting in a higher stiffness and a higher thermal expansion coefficient (Figure 7).

**Figure 8:**
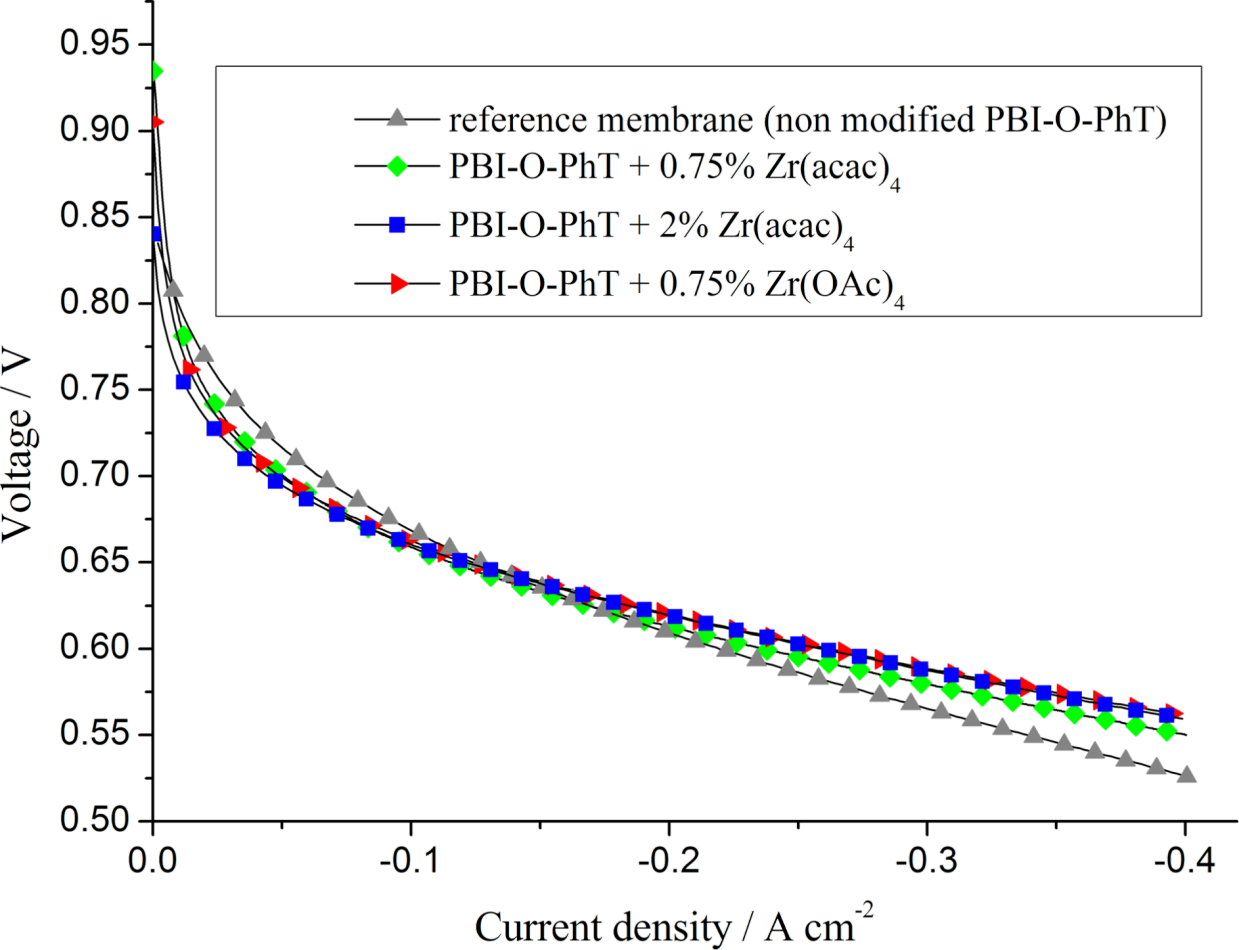
Performance of fuel cells based on PBI membranes of different types. Air is used as an oxidant, *T* = 160 °C.

**Figure 9:**
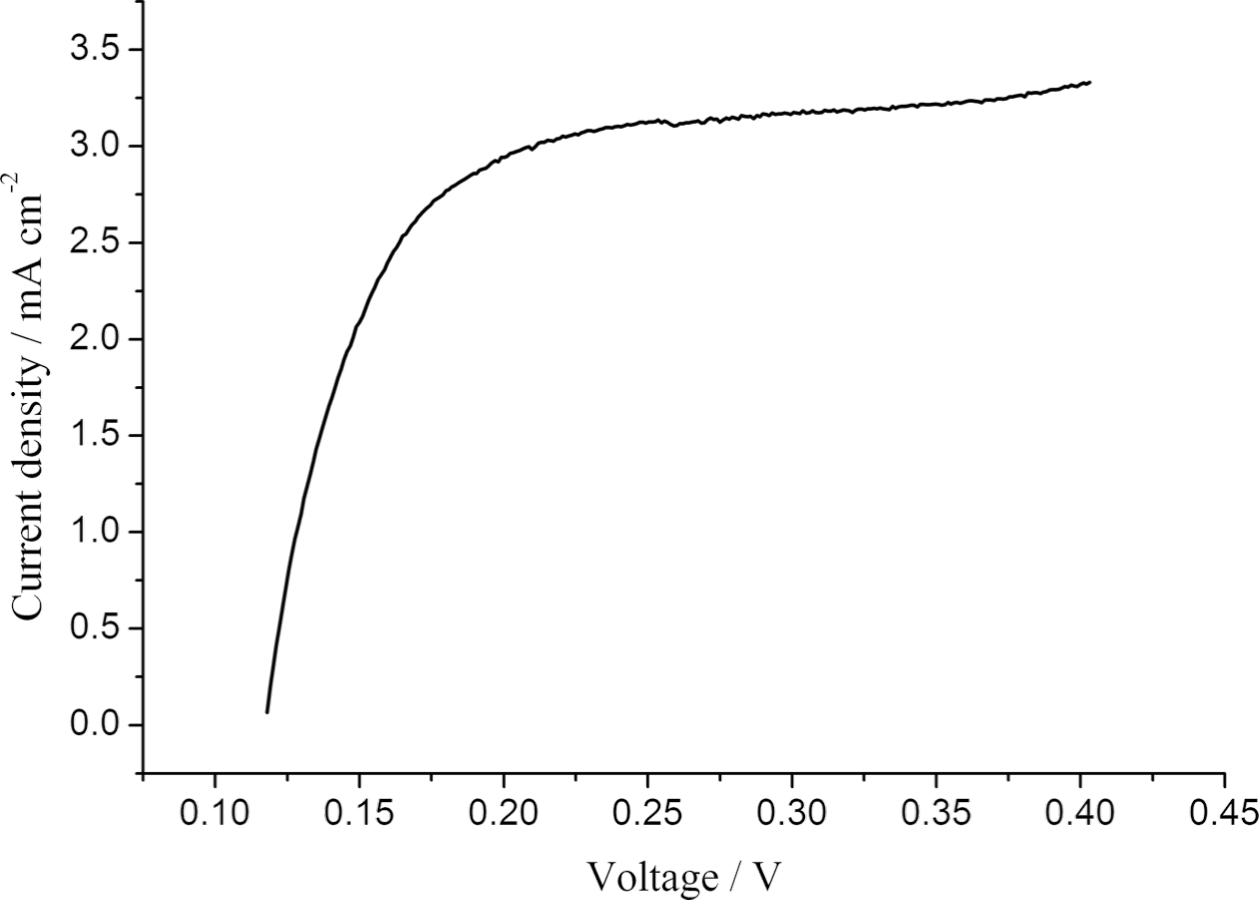
Oxidation current of hydrogen diffusing through the membrane for PBI-O-PhT with 0.75 wt % Zr(OAc)_4_. H_2_/N_2_ operation, *T* = 160 °C.

Fuel cells with all types of the Zr/PBI-O-PhT composite membranes demonstrate an enhanced performance in comparison to the pristine reference PBI-O-PhT membrane (Figure 8). High open-circuit voltages of about 900–930 mV indicate a low hydrogen permeability. Indeed, the measured hydrogen crossover-currents (Figure 9) are about 3 mA·cm^−2^ for all samples of the membranes. This is lower than the 4–5 mA·cm^−2^ reported by Neyerlin et al. [20] for PBI membranes from a commercial Celtec P-1000 MEA (BASF). Polarisation curves of fuel cells with different composite membranes demonstrate a similar behaviour. More detailed analysis has been performed by means of EIS by using an equivalent circuit with a transmission line for the approximation of impedance spectra.

**Figure 10:**
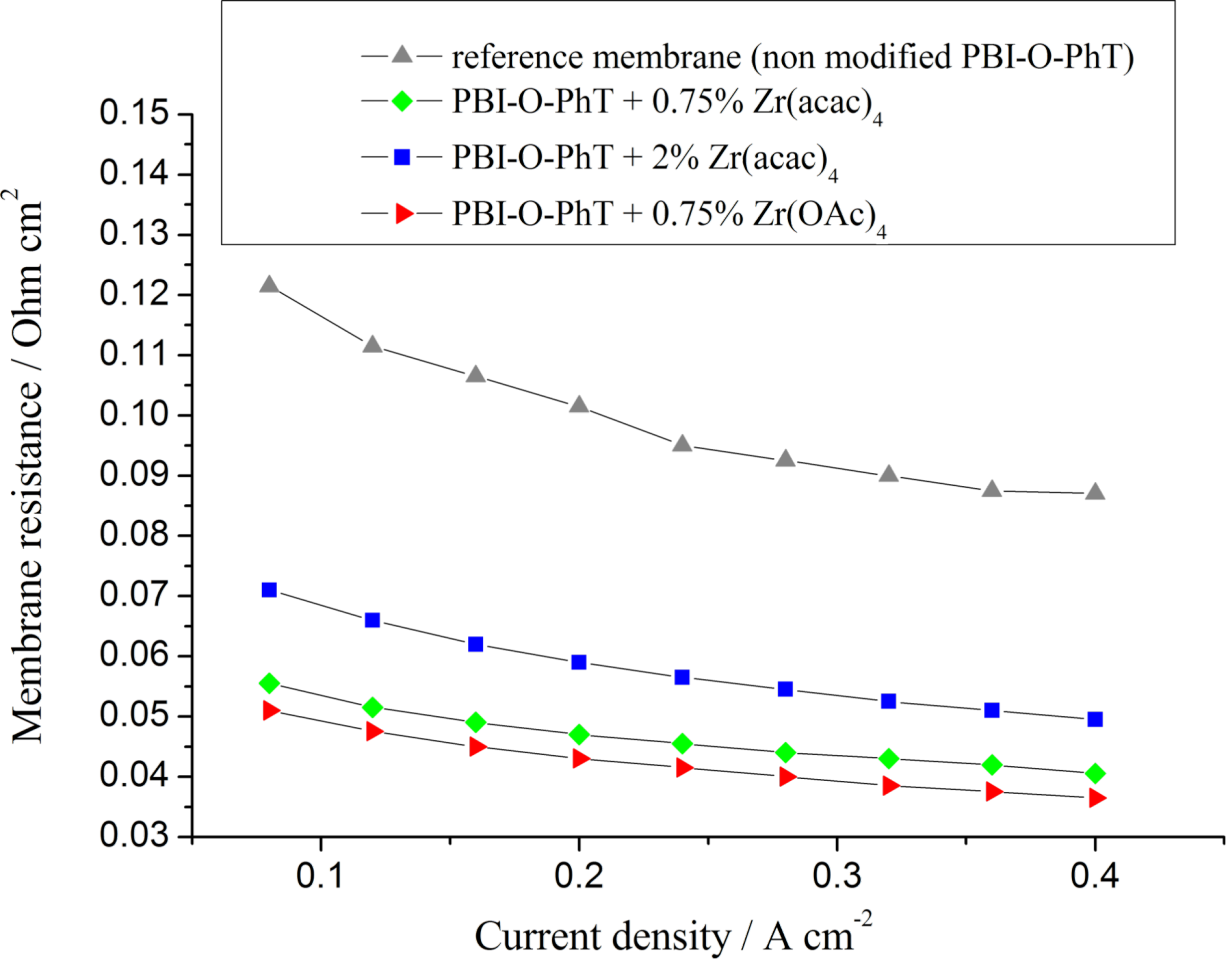
Membrane resistances as functions of the current density for fuel cells with different PBI membranes. *T* = 160 °C.

According to the impedance data, using zirconium as a crosslinking agent allows to achieve a significantly reduced membrane resistance in comparison to the non-modified reference sample (Figure 10). The membrane thickness is virtually identical for all samples (about 50 μm). That means that the conductivity of the composite membranes is noticeably higher (about 0.04 S·cm^−1^, 0.07 S·cm^−1^, and 0.1 S·cm^−1^ at 160 °C for the pristine crosslinked PBI-O-PhT reference membrane, for PBI-O-PhT with 2 wt % Zr(acac)_4_ and for PBI-O-PhT with 0.75 wt % Zr(acac)_4_ or Zr(OAc)_4_ loading, respectively). The increase of the conductivity is because of the higher uptake of PA by the composite Zr/PBI-O-PhT film. It can be explained by the dynamic nature of the zirconium crosslinks between the macromolecules and the ability of zirconium to form acidic phosphates and act as a coordination centre for PA. As shown in Table 1, the PA uptake of a membrane decreases with an increasing degree of crosslinking, so the observed resistance of membranes with 2 wt % Zr precursor loading is higher than for membranes with 0.75 wt % loading.

**Figure 11:**
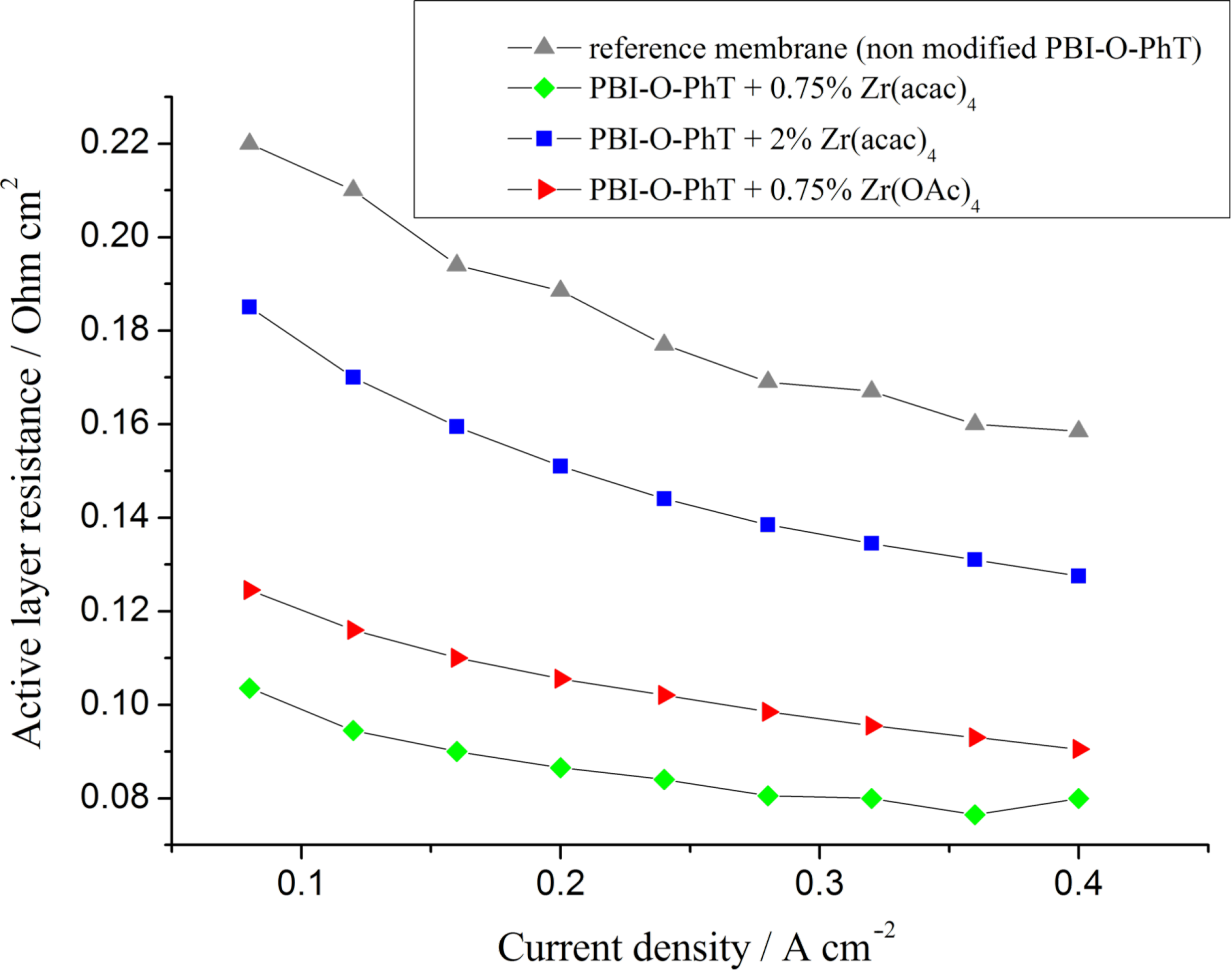
Distributed cathode active layer resistances as functions of current density for fuel cells with different PBI membranes. *T* = 160 °C.

According to data presented in Figure 11, the distributed resistances of the cathode AL are also lower in fuel cells with composite membranes containing Zr. This beahvior is expected. Since the composite films take up more PA they should release more PA into the AL of the electrodes during the break-in of the fuel cell. According to our previous study [19] the amount of acid inside the AL has a significant influence on the distributed resistance: the higher the content of PA electrolyte in the electrode AL, the lower their resistance. This way the observed active-layer resistance values correlate with the equilibrium acid-doping level of the membranes presented in Table 1.

**Figure 12:**
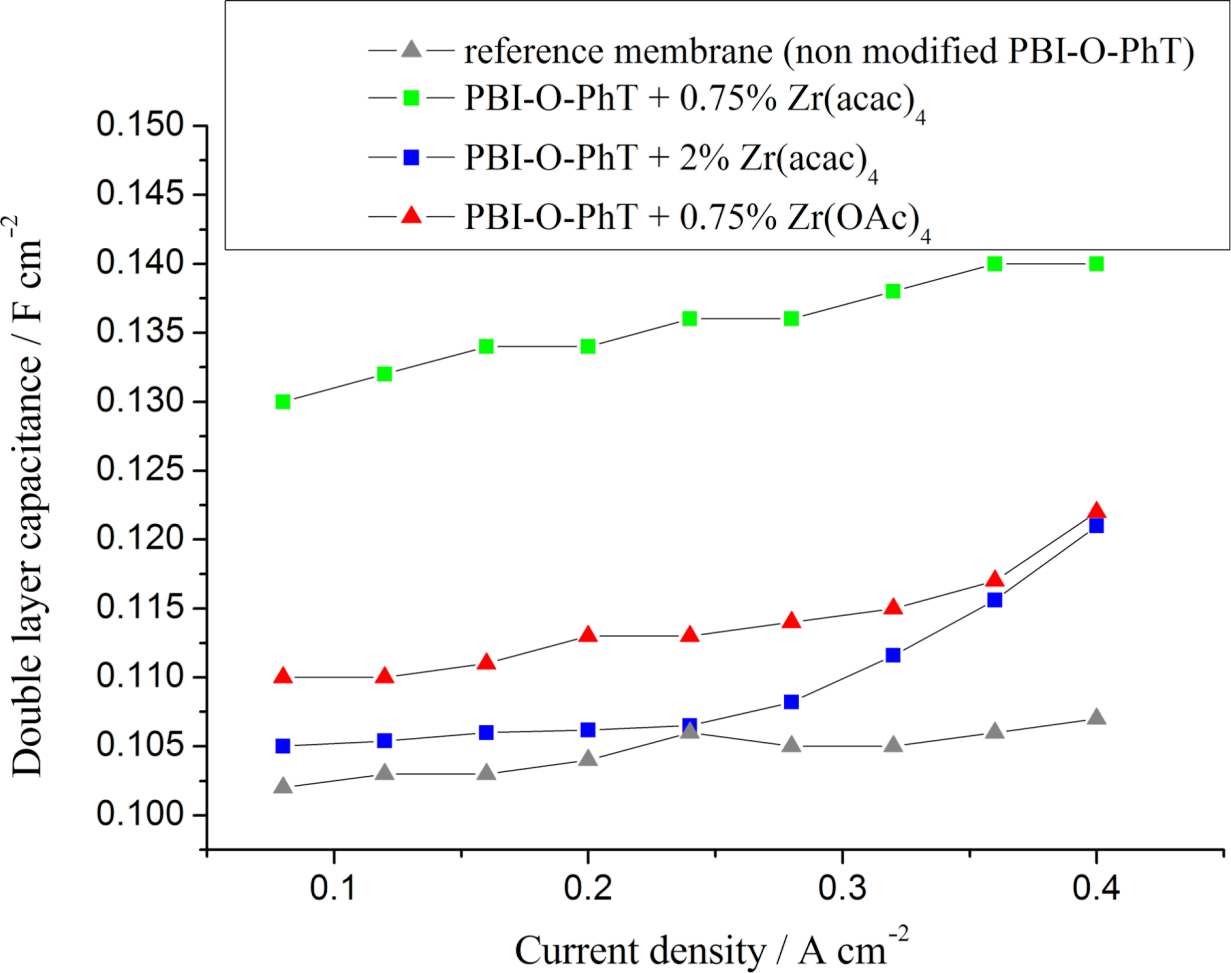
The double layer capacitance as a function of the current density for fuel cells with different PBI membranes (*T* = 160 °C). A higher capacitance indicates a higher area of interphase boundary (PA/Pt and C), which depends on the amount of PA in the active layer.

The dependence of the acid content in the electrode AL on the equilibrium doping-level of the membranes is also confirmed by the double-layer capacitance data presented in Figure 12. The capacitance values, which can be measured by means of impedance spectroscopy, depend on the boundary area between the proton- (PA) and electron- (Pt and carbon support) conducting phases and serve as a good indicator of the acid content in the AL [22–23]. The data in Figure 12 indicate that membranes with 0.75 wt % Zr(acac)_4_ release the highest amount of acid into the AL. The lowest amount of PA is in the AL of cells with the pristine reference membranes, which is in good agreement with AL resistance data (Figure 11). It is interesting that the cells with composite membranes modified by adding 0.75 wt % Zr(OAc)_4_ have lower double layer capacitance values in comparison to the cells with PBI-O-PhT membranes modified by adding the same amount (0.75 wt %) of Zr(acac)_4_. That means that membranes for which Zr(OAc)_4_ was used as a Zr precursor release less PA into the electrodes than membranes with the same loading of Zr(acac)_4_. At the same time, these membranes contain the same quite high amount of PA (430%, see Table 1). The enhanced acid-retaining properties of PBI-O-PhT + Zr(OAc)_4_-membranes may be explained by the hydrolysis of Zr(OAc)_4_ by water vapour present in the air during the film-casting process. This could result in the formation of a Zr oxide film at the surface of the membrane, and the oxide film may prevent acid from leaching out of the membrane. The fact that the acid retention properties of the PBI membranes depend on the type of Zr precursor is unexpected and needs further investigation.

**Figure 13:**
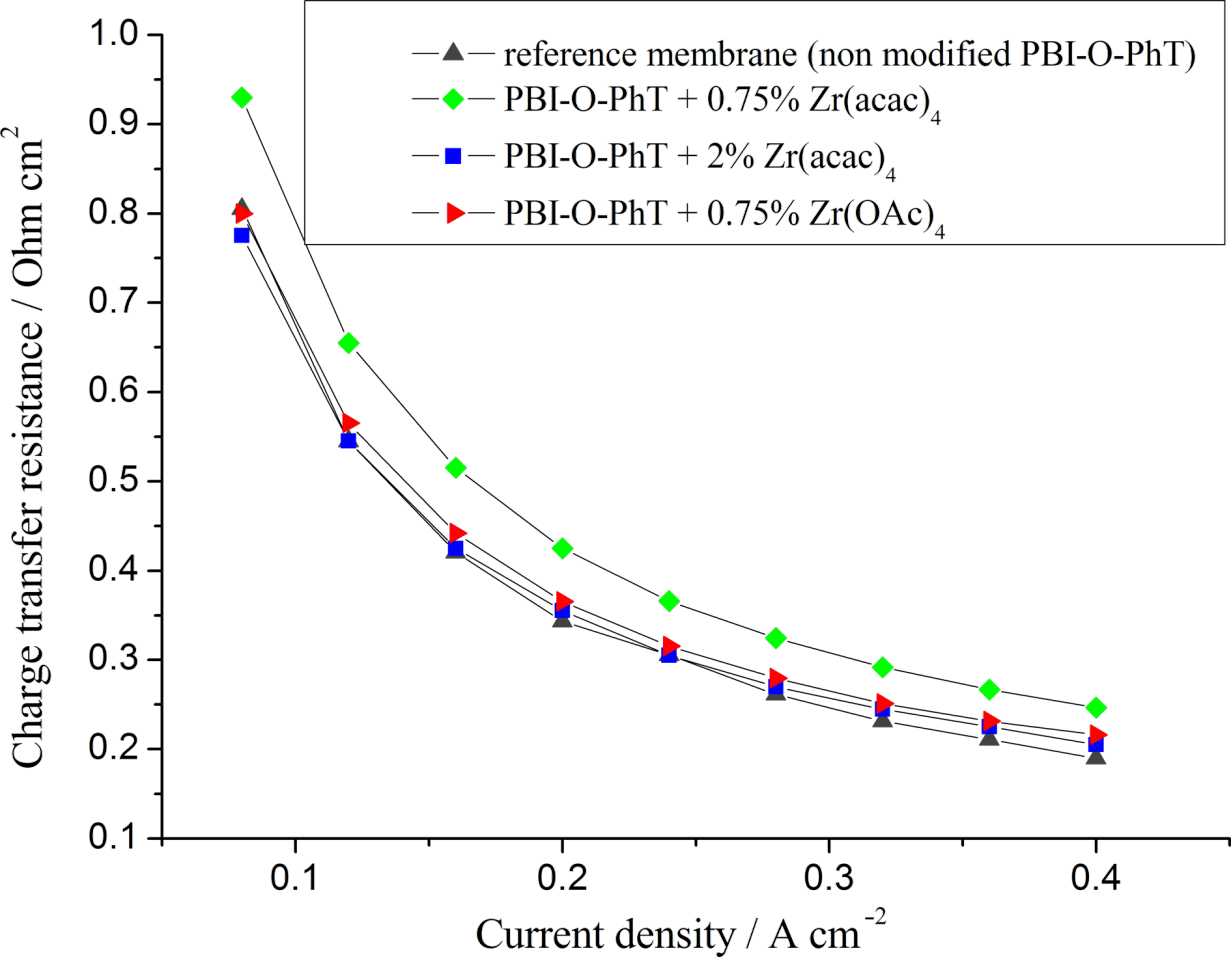
Polarization resistance (the sum of charge-transfer and mass-transfer resistances) as a function of the current density for fuel cells with different PBI membranes (*T* = 160 °C). Higher resistances indicate a lower oxygen transport in the active layer because of a higher acid content in AL.

According to [23] one cannot distinguish charge- and mass-transfer processes from a single impedance spectrum of HT-PEFC since they have similar relaxation times. Thus, the polarization resistances presented in Figure 13 account for both charge- and mass-transfer contributions. Since the electrodes in all FC tests are the same, the differences of the polarization resistance are mainly due to the redistribution of PA inside the MEA and the variation of the PA amount inside the electrode AL. An increase of this amount leads to a flooding of the AL pores and a less effective oxygen transport, which results in higher polarization-resistance values. Thus, the highest resistance is observed for FC with PBI-O-PhT membranes modified with 0.75 wt % Zr(acac)_4_, which release large amounts of acid in the AL. The PBI-O-PhT films with 0.75 wt % Zr(OAc)_4_ release lower amounts of PA, and so the polarization resistance for FC based on these membranes is lower. Yet the lowest polarization resistance is observed for FC with the pristine reference membrane since it contains and releases the lowest amount of liquid electrolyte. It is noteworthy, that the comparative analysis of the acid content of the AL by using polarization-resistance measurements is in good agreement with AL-resistance and double-layer capacitance data.

**Figure 14:**
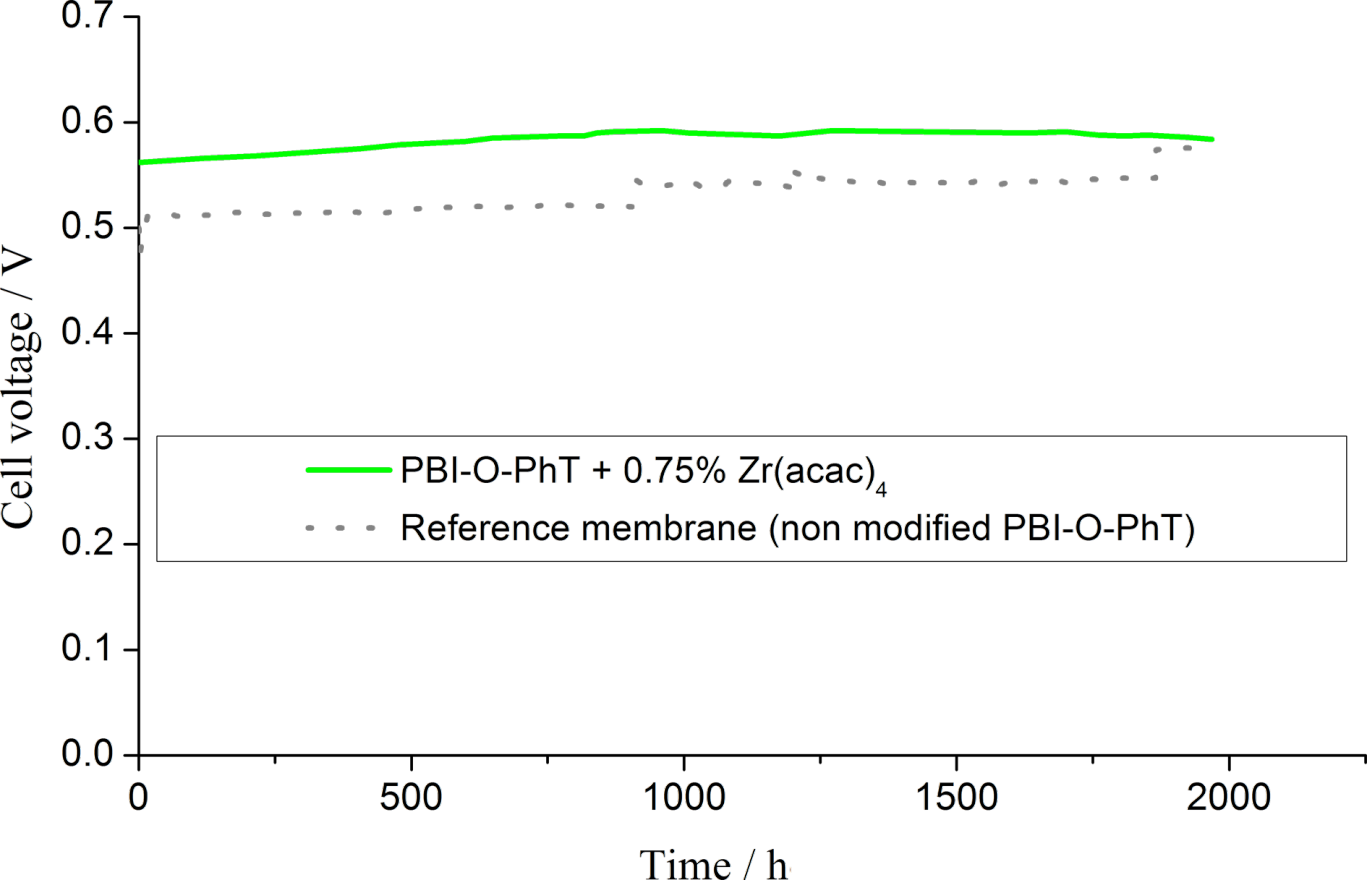
2000-hour stability test of a composite PBI-O-PhT + 0.75 wt % Zr(acac)_4_ membrane and the reference membrane in an operating fuel cell. H_2_/air, *T* = 160 °C, current density 0.4 A·cm^−2^.

According to the impedance data, PBI-O-PhT membranes modified by adding an amont of 0.75 wt % Zr precursor are good candidates for FC applications. In order to check their durability and overall chemical and thermal stability the membranes were tested in operating fuel cells for 2000 h (Figure 14). An increase in performance of about 15–20 mV due to the redistribution of PA inside the MEA and the formation of the effective boundary between the three phases (electron and proton conducting phases and gas phase) is observed during the first 1000 h. This relaxation time is noticeably higher than the typical time of about 100 hours reported in the literature as a suitable break-in period of HT-PEFC [24]. This may be attributed to the high PA amount in the composite membrane. The characteristic 1000 h may be the time needed to remove excessive acid from the AL of the electrodes. It is worth noticing that after the first thousand hours of operation, the performance of the FC with composite membranes is stable and at the same time higher than that of the pristine-membrane reference sample.

## Conclusion

Adding small amounts (0.5–2.0 wt %) of zirconium precursors (Zr(acac)_4_ or Zr(OAc)_4_) into PBI-O-PhT films and subsequent heating allows to produce composite membranes with thermally and chemically stable zirconium crosslinks. The membranes become insoluble in PA and may be used in fuel cells without any additional crosslinking. After PA doping, due to the expected dynamic nature of Zr-crosslinks, these novel composite membranes demonstrate an enhanced PA uptake providing improved proton conductivity and at the same time exhibit a favourable thermal and mechanical stability. The acid-retention ability of the composite membranes is also improved but depends on the type of the Zr precursor. The films modified with Zr(OAc)_4_ keep acid more strongly than films modified with the same amounts of Zr(acac)_4_. It is possible to manage the acid redistribution inside the MEA by varying the amount of the Zr precursor: the higher the Zr content, the better the acid-retention properties. The existence of an optimal Zr content in a PBI-O-PhT film was shown. Larger amounts of Zr lead to a lowering of the PA doping level and a lower conductivity due to a higher degree of crosslinking.
